# Cutis marmorata telangiectatica congenita révélant un lupus neonatal

**DOI:** 10.11604/pamj.2014.18.339.5013

**Published:** 2014-08-27

**Authors:** Kenza Baline, Hakima Benchikhi

**Affiliations:** 1 Service de Dermatologie et de Vénéréologie, CHU Ibn Rochd, Casablanca, Maroc

**Keywords:** Cutis marmorata telangiectatica congenita, lupus néonatal, stries érythémato-violacées livédoides, Cutis marmorata telangiectatica congenita, neonatal lupus, erythematous livedoid stripes

## Image en médecine

La cutis marmorata telangiectatica congenita (CMTC) est une anomalie vasculaire rare d'aspect clinique caractéristique. De nombreuses associations ont été rapportées avec cette malformation vasculaire, cependant, son association avec un lupus néonatal a été rarement décrite. Nous rapportons l'observation d'un nourrisson de 3 mois qui présentait des stries érythémato-violacées livédoides diffuses à tout le corps anastomosées entre elles avec un aspect marbré parcourues par de fines télangiectasies. Le diagnostic clinique d'une CMTC a été posé. Les explorations paracliniques révélaient une anémie à 9.1g/dl hypochrome microcytaire, une élévation asymptomatique des ASAT=92UI/l (≤35). Le bilan immunologique a montré des anticorps antinucléaires positifs et des auto-anticorps anti SSA/Ro positifs, les auto-anticorps anti SSB et anti RNP étaient négatifs. Le profil immunologique de la mère révélait aussi des anticorps antinucléaires, anti SSA/Ro et anti SSB/La positifs. Le diagnostic d'un lupus néonatal a été donc posé. En l'absence d'une manifestation cardiaque et devant la normalisation du bilan hématologique et hépatique, seule une surveillance clinique et biologique a été préconisée chez le nourrisson et la mère. Il s'agit d'un cas rare très particulier de CMTC ayant révélé un lupus néonatal (seulement 7 cas décrits dans la littérature). Notre cas appuie l'idée avancée par certains auteurs qui s'accordent à interpréter la CMTC au-delà d'une simple association fortuite avec un lupus néonatal mais qu'elle doit plutôt s'intégrer dans le spectre des manifestations cutanées de cette pathologie.

**Figure 1:**
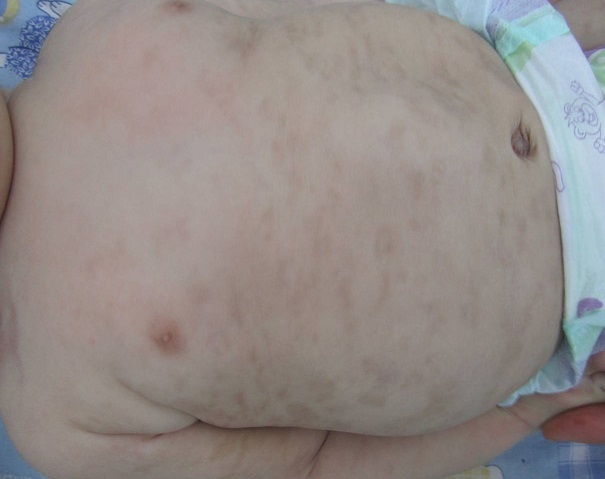
Stries érythémato-violacées livédoides

